# Consortium-Based Science: The NIEHS’s Multipronged, Collaborative Approach to Assessing the Health Effects of Bisphenol A

**DOI:** 10.1289/ehp.1205330

**Published:** 2012-09-25

**Authors:** Linda S. Birnbaum, John R. Bucher, Gwen W. Collman, Darryl C. Zeldin, Anne F. Johnson, Thaddeus T. Schug, Jerrold J. Heindel

**Affiliations:** 1 National Institute of Environmental Health Sciences (NIEHS), National Institutes of Health (NIH), Department of Health and Human Services (DHHS), Research Triangle Park, North Carolina, USA; 2 Division of the National Toxicology Program,; 3 Division of Extramural Research, and; 4 Division of Intramural Research, NIEHS, NIH, DHHS, Research Triangle Park, North Carolina, USA; 5 MDB Inc., Research Triangle Park, North Carolina, USA; 6 Division of Extramural Research, Cellular, Organs and Systems Pathobiology Branch, NIEHS, NIH, DHHS, Research Triangle Park, North Carolina, USA

**Keywords:** bisphenol A, consortium-based research, endocrine disruptor, low dose, NIEHS

## Abstract

Background: Bisphenol A (BPA) is a high production volume chemical used to make polycarbonate plastic and is found in many consumer products. Some studies using animal models have suggested that BPA exposures may have adverse health effects. However, research gaps have precluded a full understanding of the effects of BPA in humans and engendered controversies surrounding the chemical’s potential toxicity.

Objectives: The National Institute of Environmental Health Sciences (NIEHS) and National Toxicology Program (NTP) have developed an integrated, multipronged, consortium-based approach to optimize BPA-focused research investments to more effectively address data gaps and inform decision making.

Discussion: NIEHS/NTP BPA research investments made over the past 4 years include extramural research grants, establishment of a BPA Grantee Consortium, intramural research activities on BPA’s mechanisms of action, the launch of two clinical studies and an occupational study, development of a round-robin experiment to validate BPA measurements in human serum, and, in collaboration with the Food and Drug Administration (FDA), formation of a consortium to design and execute a chronic toxicity study of BPA in rats. The NIEHS’s new consortium-based approach has led to more integrated, collaborative efforts and should improve our ability to resolve controversies over the potential human health effects of exposures to low levels of endocrine-active agents.

Bisphenol A (BPA) is used to produce polycarbonate plastics, epoxy resins, and other products. Manufacturers produce > 8 billion pounds of BPA every year, making it one of the most common industrial chemicals produced worldwide (Rubin 2011). Plastics made with BPA are used in many consumer products, including food and beverage containers, toys, eyeglasses, computers, kitchen appliances, and medical equipment. Epoxy resins containing the chemical are used in dental work and in metal coatings for food cans, pipes, cars, dairy equipment, office equipment, and other metal products. BPA is also used in the production of certain flame retardants and as a color developer in some thermal receipt paper.

BPA has been detected in air, soil, water, landfill leachate, and the human body. The primary source of human exposure to BPA is thought to be through the diet. The chemical has been shown to leach into foods and beverages from food packaging and reusable containers (Von Goetz et al. 2010). People also may be exposed to BPA through skin contact, inhalation, dental fillings, and occupational exposures. BPA has been found in human serum, milk, saliva, urine, and amniotic fluid (Vandenberg et al. 2009, 2010, 2012).

The ubiquity of BPA in the environment and in the human body has led to concerns about adverse health effects. BPA’s chemical structure [see Supplemental Material, Figure S1 (http://dx.doi.org/10.1289/ehp.1205330)] allows it to fit into the estrogen receptor binding pocket, and BPA is considered to act as an endocrine disruptor. BPA binds to both nuclear and cell membrane estrogen receptors; at higher levels, BPA acts as an androgen receptor antagonist and interacts with the thyroid receptor (Vandenberg et al. 2009). Animal and human research has associated BPA with many health problems including infertility, weight gain, behavioral changes, early-onset puberty, prostate and mammary gland cancers, cardiovascular effects, and diabetes.

## Impetus for the NIEHS’s Strategic Focus on BPA

More than 800 studies were published on the health effects of BPA between the mid-1990s and the mid-2000s. Many showed some form of toxicity, but critical data gaps and uncertainties led to discussion about how the research should be interpreted. In response to increasing concerns about BPA toxicity, the National Institute of Environmental Health Sciences (NIEHS) began developing a targeted BPA research program in the mid-2000s.

As a first step, the NIEHS organized a workshop to examine the body of evidence related to BPA. The resulting report, known as the “Chapel Hill consensus statement” (vom Saal et al. 2007), along with five review articles (Crain et al. 2007; Keri et al. 2007; Richter et al. 2007; Vandenberg et al. 2007; Wetherill et al. 2007), concluded that human exposure to BPA is widespread and that the adverse health effects observed in animal studies raised significant concerns about the potential for similar effects in humans. The report also outlined research gaps and needs.

Around the same time, the National Toxicology Program (NTP) Center for the Evaluation of Risks to Human Reproduction (CERHR) convened an expert panel to examine BPA research related to human reproduction and development. Based largely on the panel’s assessment (Chapin et al. 2008), the NTP (2008) reported “negligible concern” for reproductive effects in nonoccupationally exposed adults and “minimal concern” for occupationally exposed workers, but identified “some concern” for effects on the brain, behavior, and prostate gland in fetuses, infants, and children at current levels of human BPA exposure.

In 2010, the Food and Drug Administration (FDA), which has regulatory authority over many consumer and medical products containing BPA, issued a statement expressing its agreement with the NTP’s conclusion for some concern about the effects of BPA on the brain, behavior, and prostate gland of fetuses, infants, and children (FDA 2012). The FDA also identified substantial uncertainties in BPA research findings and their implications for human health. The statement called for further research to address these uncertainties; in the interim, the FDA encouraged consumers and industry to take “reasonable steps” to reduce human exposure to BPA, particularly among infants. In March 2012, the FDA denied a petition from the Natural Resources Defense Council to revoke regulations permitting the use of BPA in food-contact materials, citing insufficient evidence that BPA is unsafe at current levels of human exposure (Dorsey 2012).

The Chapel Hill consensus statement, the NTP-CERHR monograph, and the FDA’s statements have helped to focus the field by identifying gaps in BPA research and highlighting concerns that need to be addressed to move the field forward. Key sources of uncertainty that have been identified include *a*) absent or inconsistent data on dose response, including low-dose effects and nonmonotonic dose–response behaviors; *b*) pharmacokinetics across species and the lifespan; *c*) differences between the sexes; *d*) routes and extent of human exposures; *e*) sensitive windows of exposure; *f*) mechanism(s); and *g*) specific disease end points. In addition, these reports cited difficulties in extrapolating data from animals to humans and in comparing results from studies compliant with good laboratory practices (GLP) and those initiated by academic investigators.

## NIEHS BPA Research Program

**Figure 1:**
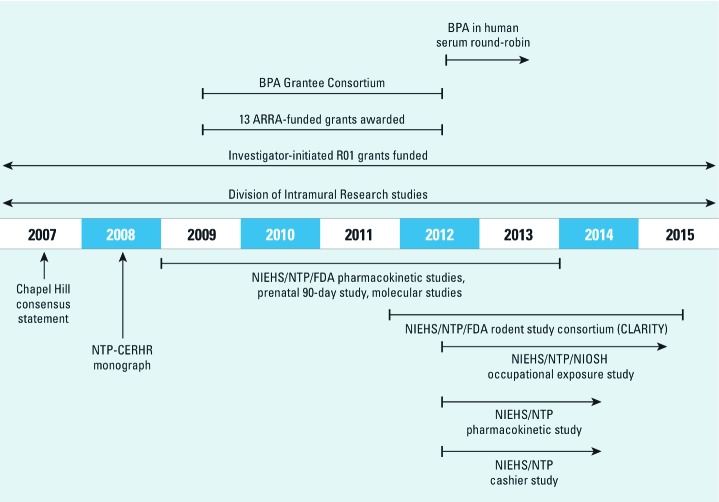
Elements of the NIEHS BPA research program. NIOSH, National Institute for Occupational Safety and Health.

Because much about the potential health effects of BPA remained unknown, the NIEHS determined that a strategic research investment in BPA was warranted to better inform risk assessments for the ubiquitous chemical (Maffini et al. 2011). To that end, in 2009 the NIEHS launched a multipronged research program designed to fill remaining data gaps and resolve controversies about the design and interpretation of BPA toxicity studies. The plan included significant extramural and intramural research investments, as well as intra- and interagency coordination and collaboration. Elements of the NIEHS’s BPA research program are described below and summarized in Figure 1. The extramural program’s efforts to address specific BPA research challenges are summarized in Supplemental Material, Table S1 (http://dx.doi.org/10.1289/ehp.1205330). The NIEHS established a transagency working group with members from each of its research divisions (intramural research, extramural research, and NTP) to facilitate coordination of these efforts.

*Extramural research grants and the BPA Grantee Consortium.* By 2008, the NIEHS had funded 39 grants to assess BPA toxicity, all of which were investigator initiated. In 2009, the NIEHS awarded 10 BPA-focused Grand Opportunity grants [see Supplemental Material, Table S2 (http://dx.doi.org/10.1289/ehp.1205330)] and 3 Challenge Grants using funds made available through the American Recovery and Reinvestment Act (ARRA). The ARRA-funded grants were specifically targeted at addressing data gaps identified by the FDA, including measurement of BPA in serum, evaluation of dose response, measurement of disease end points, replication of results, assessment of gender-related differences, and use of overlapping end points among animal studies and between animal and human studies.

To work toward a comprehensive, integrated assessment of the health effects of BPA, in 2009 the NIEHS brought existing BPA grantees together with the new ARRA-funded grantees into a BPA Grantee Consortium. This consortium includes > 40 researchers. Members have gathered three times in person and met approximately once per month via conference call from late 2009 to the present. This frequent communication has facilitated close collaboration and free exchange of ideas, materials, tissues, and data. The BPA Grantee Consortium has several specific activities and areas of focus that are described below.

Improved integration of studies. Efforts to extrapolate results between animal and human studies have been complicated by disconnects among the doses and end points investigated. Related research gaps include the mechanisms and speed with which BPA is metabolized and excreted in the human body and whether humans and animals process BPA in the same way.

To fill research gaps, resolve discrepancies, and produce results that will be more interpretable across studies, consortium members have worked to establish consistency in the models, approaches, doses, and end points used across the spectrum of NIEHS-funded BPA research. In addition, grantees were required to use BPA provided by the NIEHS to ensure quality control and consistency. Studies using mice, rats, rhesus monkeys, and humans were coordinated to develop an integrated assessment of BPA metabolism across species; some of these results have already been published (NIEHS 2012). In addition, the NIEHS/NTP clinical pharmacokinetic study will provide information about the rates of BPA metabolism in human volunteers. The results of that study, combined with the contributions from the BPA Grantee Consortium, will further clarify how data from different animal models may be used to understand and predict health risks in humans.

Round-robin assessment of BPA in human serum. The ability to accurately measure exposure to BPA is critical to assessing the chemical’s health effects. Measuring BPA in urine is generally considered the most reliable indicator of BPA exposure because it integrates exposure over a recent time period, whereas BPA concentrations in blood are thought to reflect only current exposures due to the chemical’s short half-life and evidence that BPA does not bioaccumulate. However, serum measurements are currently the most meaningful way to assess levels of unconjugated BPA (also known as free BPA), which is the form considered to be more biologically active because it can bind to estrogen and other nuclear receptors (Ye et al. 2011). This is an area that requires further investigation because some researchers posit that rapid processing of BPA in the liver during “first pass” metabolism results in only conjugated (and thus biologically inactive) BPA entering the bloodstream, precluding BPA from causing disease. However, > 30 reports have found human blood levels of BPA in the range of 0.1–4.0 ng/mL (Taylor et al. 2011; Vandenberg et al. 2010). Although, in some cases consistent results have been reported across studies, these findings have spurred debate in the scientific community about the possibility for measurements to be compromised by contamination. For example, small amounts of BPA may leach into samples from syringes, containers, tubing, or even water used in experiments. In addition, instruments used to measure levels of BPA may be incorrectly calibrated.

The NIEHS provided supplemental funding to address these controversies through a series of round-robin experiments. Members of the BPA Grantee Consortium from five laboratories are measuring BPA levels in a shared collection of uncontaminated blood samples and samples spiked with various amounts of free and conjugated BPA (BPA glucuronide, BPA sulfate, and coded samples were provided by the NIEHS to ensure quality control). Participating laboratories are also assessing potential sources of contamination and error in their research protocols. Once the experiments produce comparable results and show how to eliminate contamination and other errors, the protocols will be shared for others to use.

Characterization of low-dose effects. Another factor contributing to uncertainty in BPA research stems from inconsistencies in the characterization of low-dose effects. Studies in both animals and humans have indicated effects of BPA both at low (nanomolar or lower) and high (micromolar or higher) doses, often with fewer effects at mid-level doses (Vandenberg et al. 2012). Such nonmonotonic (U-shaped) effects curves may reflect BPA activity in different systems, with low doses causing effects in the endocrine system and high doses potentially causing effects in another organ system. Other studies have reported effects at very low doses but have not included higher doses, making the dose–response relationship difficult to characterize.

In addition, the definition of “low dose” for BPA exposure has led to some debate within the research community. The 2008 NTP-CERHR report (NTP 2008) defined a “low” dose as ≤ 5 mg/kg body weight per day. However, members of the scientific community have suggested that the “low-dose” designation be based on concentrations administered to animals that produce blood levels in the range of those measured in human tissues and fluids (0.1–4.0 ng/mL) (Vandenberg et al. 2012).

The BPA Grantee Consortium provides a forum for discussion about low-dose effects, and through their individual research efforts, consortium members have investigated the effects of low doses of BPA in mice, sheep, and monkeys on multiple disease end points. In addition, grantees of the CLARITY-BPA program (Consortium Linking Academic and Regulatory Insights on BPA Toxicity) are collaborating on a GLP-compliant toxicity study over a wide range of administered BPA doses in rats.

Improving precision for measuring BPA exposures. Human epidemiological studies provide valuable information about the internal levels of human exposure to chemicals and resulting health impacts. Although the National Health and Nutrition Examination Survey (NHANES; Calafat et al. 2008; Stahlhut et al. 2009)—a statistically based sampling of the American population conducted every 2 years—and several birth cohorts, such as the Center for the Health Assessment of Mothers and Children of Salinas (CHAMACOS) study cohort (Castorina et al. 2010), have contributed useful data on BPA exposure and impacts, the design and methodology of these studies make it difficult to draw conclusions from them about BPA’s health effects (Stahlhut et al. 2009). One reason is that most epidemiological studies have collected urine samples from participants based on convenience rather than at specific time intervals or times of day, making it difficult to compare levels of BPA exposure among samples and participants. In addition, the mechanisms and speed with which the body processes BPA after oral exposure are poorly understood, making it difficult to assess BPA exposure based on measures of BPA conjugates in urine. Potential contamination of samples from laboratory equipment or water containing BPA is also a concern (Vandenberg et al. 2010).

The BPA Grantee Consortium has aimed to address these issues by developing standard protocols and approaches for measuring BPA exposure in humans. Future epidemiological studies can be optimized for BPA research by including serum samples in the study protocol, standardizing time intervals for sampling, and ensuring that all equipment is properly calibrated and BPA free. The round-robin assessment of BPA in serum and the NIEHS/NTP pharmacokinetic study will provide further insights to inform approaches to detecting BPA exposure levels in humans.

Comprehensive assessments in targeted areas. Consortium members established subgroups to characterize and coordinate research in the following areas: biomonitoring, pharmacokinetics, reproductive effects, cancer, metabolic effects, neurobehavioral effects, low-dose effects, and immune effects. Each subgroup is surveying results published since 2007 and adding data from consortium members’ ongoing studies to assess the strength of the evidence in these areas of investigation. The results of these literature-based assessments are expected to be reported as a series of manuscripts in early 2013.

Third-party evaluation. NIEHS has enlisted the aid of the Battelle Centers for Public Health and Evaluation (Durham, NC) for an independent assessment of how ARRA-funded research investments have affected the direction and outcomes of the NIEHS’s BPA research program.

*CLARITY-BPA program.* Findings from studies conducted in accordance with GLP are often used to inform regulatory decision making for potentially harmful chemicals. Several past animal studies designed in accordance with GLP have examined the toxicity of BPA (e.g., Tyl et al. 2002, 2008). Although these studies have made valuable contributions, some gaps remain with regard to the exposures and end points that have been investigated. For example, none of the GLP-compliant studies on chronic BPA exposure have included developmental exposure with direct, rather than lactational, exposure of pups. In addition, none of these studies have evaluated the internal doses of BPA that are associated with health effects, nor have they evaluated several disease end points that, although not traditionally included in GLP studies, have been linked to BPA exposure by other animal and human studies (Myers et al. 2009).

To validate previous findings and address remaining research gaps, the NIEHS/NTP has collaborated with the FDA to establish the CLARITY-BPA program to advise the design and execution of a comprehensive GLP-compliant study of BPA toxicity in rats. The study, which began in summer 2012, is being conducted at the FDA’s National Center for Toxicological Research (NCTR). The strain of animal, animal diet and housing conditions, numbers of animals, dosing regimen, and route of exposure to BPA will be tightly controlled.

To enhance the capacity of this study to provide useful data on a broad array of relevant disease end points, a consortium of researchers has been formed. This consortium includes 12 grantees who have proposed hypothesis-driven mechanistic studies that have been accommodated within the study design [see Supplemental Material, Table S3 (http://dx.doi.org/10.1289/ehp.1205330)]. Grantees have also been involved in determining the protocols for the overall study. Although all the animals will be housed and dosed at the NCTR, grantees will have access to tissue samples and animals from the GLP-compliant study to investigate specific disease end points, many of which are not typically assessed in studies carried out according to standard regulatory guidelines. To protect the integrity of the GLP study, all researchers will be blinded to the BPA exposure levels of the animals and tissues, with identifying codes housed at the NTP. In addition, the NTP will provide advice as needed for statistical analyses and will provide for a common data repository [Chemical Effects in Biological Systems (CEBS); Waters et al. 2008].

The consortium represents an unprecedented approach to conducting GLP-compliant research by bringing researchers and regulators together during the planning stage to ensure that results will be maximally useful for risk assessment and regulatory decision making. The grantees and FDA representatives, along with coordinators from the NIEHS/NTP, held their first in-person meeting in March 2012. This collaboration is expected to produce a robust and valuable body of work on the effects of BPA in rats, a key animal model in toxicity testing.

*Intramural research activities.* Illuminating how BPA interacts with receptors in the body is an important aspect of understanding the chemical’s toxicity. The NIEHS Division of Intramural Research (DIR) has made significant investments in studying the patterns of response and molecular mechanisms of BPA and other potentially estrogenic chemicals. In experiments using mice, Hewitt and Korach (2011) used microarrays to compare BPA with other estrogenic compounds and found that BPA could be classified as a weak estrogen, similar to estriol. DIR researchers are conducting research on BPA’s interactions with estrogen receptors α and β in human cell lines, developing *in vitro* and *in vivo* screening tools to gauge estrogenicity that could be used to test BPA, and also collaborating with extramural grantees who are investigating BPA in non-human primates. These efforts aim to further advance scientists’ ability to evaluate modes of action and mechanisms of BPA and other potentially estrogenic chemicals.

*NIEHS/NTP pharmacokinetic study.* Consumption of canned foods and beverages is thought to be a major route of human exposure to BPA, and Teeguarden et al. (2011) detected measurable blood levels of total (free and conjugated) BPA in a small fraction of individuals consuming a diet heavy in canned foods and juices. However, only one study has directly examined the kinetics of BPA metabolism and elimination in human volunteers given measured amounts of BPA orally (Völkel et al. 2002), and that study used analytical methods that were less sensitive than current techniques (Vandenberg et al. 2010). Because our understanding of human pharmacokinetics remains limited, the NTP and the NIEHS Clinical Research Unit have developed a protocol to investigate BPA metabolism and excretion in humans after oral ingestion. To support development of a refined physiologically based pharmacokinetic model, up to 50 healthy adult volunteers will be administered a low oral dose of deuterated BPA (d-BPA; 100 µg/kg body weight). Blood (starting at 10 min) and urine samples will be collected for 5 days following dosing to measure d-BPA and its conjugates. By assessing how the body processes and excretes BPA, the study aims to inform investigations of human exposure to BPA as well as the chemical’s potential toxicity. Combined with extramural research on BPA pharmacokinetics in animal models, the study will further elucidate how BPA is processed in the body and will help improve researchers’ ability to integrate animal and human studies.

*NIEHS/NTP cashier study.* Another source of uncertainty regarding human exposure to BPA is the potential role of sources of exposure other than diet. Because BPA is used in numerous consumer products and industrial processes, inhalation and absorption through the skin have been proposed as potentially significant routes of exposure (Liao and Kannan 2011). For example, Von Goetz et al. (2010) proposed that people may inhale or ingest BPA through house dust, or inhale it through smoke from cigarettes with filters containing BPA. People may also absorb BPA through the skin when they touch objects containing the chemical. Although these sources of BPA exposure have not been well characterized, they could pose a greater health risk than ingestion because BPA that is inhaled or absorbed dermally may spend more time circulating through the bloodstream in unconjugated form than does BPA that enters the body through the oral route, which is subject to first-pass elimination.

BPA is commonly used in thermal receipt paper, and although many people touch receipts regularly, cashiers handle them more frequently than most people. The NIEHS/NTP cashier study will measure BPA and BPA conjugates in cashiers’ blood and urine samples before and after their work shifts. The study is expected to yield insights about the degree to which thermal receipt paper contributes to BPA exposure, although it will not determine whether the route of exposure is dermal or oral.

*NIEHS/NTP/National Institute for Occupational Safety and Health (NIOSH) occupational exposure study.* Workers who directly handle BPA where BPA is produced or processed may be exposed to significantly higher levels of BPA than the general population (Wang et al. 2012). The NIEHS/NTP and NIOSH have developed a study protocol to assess the routes and levels of exposure among such workers. In the study, urine samples from 120 workers, as well as samples of BPA in the air and on workers’ hands during their work shifts, will be collected and analyzed. The study aims to evaluate the levels of BPA exposure among occupationally exposed people and to identify factors contributing to occupational exposures.

## Discussion

The NIEHS supports basic and translational research to understand the role of environmental exposures in human disease and dysfunction. Our focus is on improving human health. Although investigator-initiated research is critical to this mission, in some cases a more strategic approach is warranted that stretches beyond—and maximizes the impacts of—individual grants. In the case of BPA, the hundreds of publications leading up to 2007 had not produced the necessary data risk assessors needed to inform conclusions and policy decisions about the health effects of BPA. To address the uncertainties and concerns surrounding BPA research, the NIEHS developed an unprecedented, comprehensive research program that combines extramural grant funding with targeted intramural research efforts and overarching structures for collaboration to fill research gaps, solve controversies, and provide results that can inform regulatory decision making.

These efforts are ongoing, but several outcomes are already clear. First, the approach has yielded important research insights. NIEHS grantees and intramural researchers have published > 100 papers since January 2010 that offer new data on health effects of BPA for a wide range of doses and disease end points in both humans and animal models.

In addition, the NIEHS’s approach has demonstrated the value of workshops and collaboration in helping to focus a field, identify research needs, and resolve controversies. [For a summary of BPA-focused workshops organized by the NIEHS, see Supplemental Material, Table S4 (http://dx.doi.org/10.1289/ehp.1205330).] The 2007 Chapel Hill consensus statement (vom Saal et al. 2007) and 2008 NTP-CERHR report (NTP 2008) were instrumental in pinpointing critical research needs and spurred the institute to make targeted research investments. Since then, the BPA Grantee Consortium and the CLARITY-BPA program have successfully used workshops to facilitate discussion among members of the BPA research community and develop shared solutions to address difficult research problems.

The BPA Grantee Consortium has demonstrated that members of the scientific community are eager to work together—even if it means adjusting their own approaches—in order to increase the collective impact of their work. Although unsolicited grantees were not officially funded as part of a formal consortium, most wholeheartedly embraced the consortium’s goals. After the establishment of the BPA Grantee Consortium, the NIEHS developed two other research consortia—the Engineered Nanomaterials Grand Opportunity consortium, aimed at evaluating the health effects of exposure to nanomaterials, and the Deepwater Horizon Research Consortium, developed to coordinate research on the environmental health impacts of the 2010 *Deepwater Horizon* oil spill. Together, these consortia represent a new wave of collaboration in extramural research, often in concert with intramural efforts at the NIEHS. BPA grantees willingly shared technologies, data, and end points; animal researchers and epidemiologists were excited by the opportunity to work together to inform their research approaches and develop end points that could be measured in both animal and human studies. In addition, those applying for Grand Opportunity funding agreed to make small changes to their protocols to provide data needed to fill research gaps. A detailed assessment of the results of the BPA Grantee Consortium, due to be completed in 2013, will document successes of this approach and identify areas for improvement.

Finally, the NIEHS’s BPA research program has received strong support from divisions across the NIEHS and has been embraced by other federal agencies. Strong intra- and interagency collaboration will continue to be critical to the success of the NIEHS’s BPA research investments.

The NIEHS’s multipronged, collaborative approach to BPA research can provide lessons for other areas of environmental health. Future studies on environmental contaminants may benefit from an early focus on identifying data gaps and on collaborative efforts to confront controversies (or prevent them before they arise). Fractured, uncoordinated research efforts can leave significant unanswered questions, impeding progress and making it difficult for risk assessors and regulators to interpret findings. In the future, perhaps earlier investments to identify needs and coordinate research efforts can save time and money, as well as improve our ability to protect human health.

## Supplemental Material

(1.6 MB) PDF
