# Supplementary material for: Consortium-Based Science: The NIEHS’s Multipronged, Collaborative Approach to Assessing the Health Effects of Bisphenol A
Source: Environ Health Perspect. 2012 Sep 25;120(12):1640–4. doi: 10.1289/ehp.1205330 (PMC3548284; doi:10.1289/ehp.1205330)
Supplement: (1.6 MB) PDF [file ehp.1205330.s001.pdf]

Supplemental Material

Consortium-Based Science: The NIEHS's Multipronged, Collaborative Approach to Assessing the Health Effects of Bisphenol A

Linda S. Birnbaum, John R. Bucher, Gwen W. Collman, Darryl C. Zeldin, Anne F. Johnson, Thaddeus T. Schug, and Jerrold J. Heindel

Table of Contents

Figure S1. Chemical structures of bisphenol A (4,4'-(propane-2,2-diyl)diphenol) and estradiol (17B-estra-1,3,5(10)-triene-3,17-diol)……………………………………………………………………………3

Table S1. Extramural NIEHS research investments aimed at filling research gaps and addressing sources of uncertainty regarding the health effects of BPA…………………………………..4

Table S2. BPA-focused Grand Opportunity grants supported by funding from the American Recovery and Reinvestment Act (ARRA)……………………………………………………………………..6

Table S3. BPA research supported by CLARITY study……………………………………………………………………7

Table S4. BPA-focused workshops organized by NIEHS…………………………………………………………………8

Figure S1. Chemical structures of bisphenol A (4,4′-(propane-2,2-diyl)diphenol) and estradiol (17B-estra-1,3,5(10)-triene-3,17-diol).

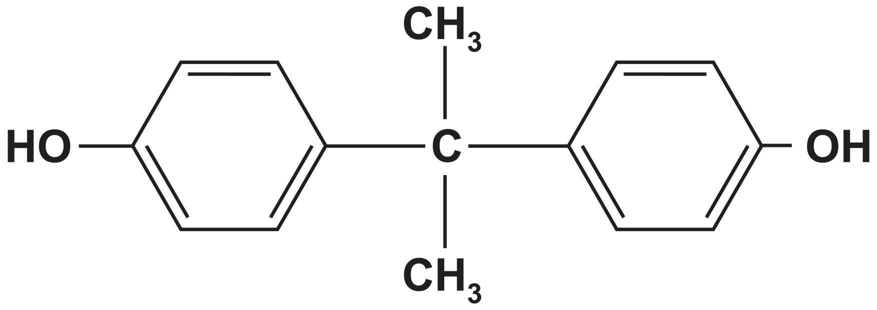

Bisphenol A

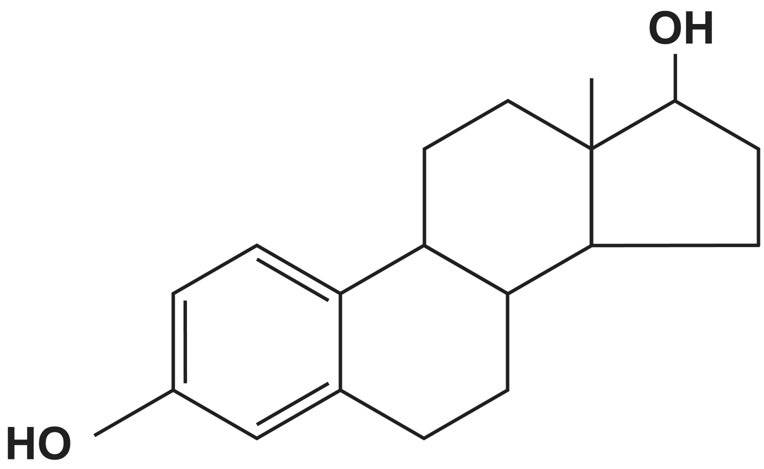

Estradiol

Supplemental Material, Table S1. Extramural NIEHS research investments aimed at filling research gaps and addressing sources of uncertainty regarding the health effects of BPA.

Research Challenge	NIEHS Research Investments
Lack of consistency in models, approaches, routes of exposure, doses, and endpoints.	Developed consortium to establish standard approaches and share technologies, materials, tissues, and data.
Persistent data gaps due to lack of communication among researchers.	Held bimonthly working group teleconferences and yearly grantee meetings to share ideas and results.
Difficulty extrapolating between animal and human studies.	Facilitated communication among researchers studying humans and animal models to establish consistent doses and endpoints across species.
Questions about accuracy and reliability of measurements of BPA levels in humans (biomonitoring).	Conducted round robin experiments to refine research protocols and eliminate errors; synthesized BPA standards and established internal standards for use in BPA measurements.
Gaps in understanding of routes of human exposure and links between route of exposure and effects.	Requested grantees assess multiple routes of exposure and measure blood levels of BPA to relate to exposure and effects.
Inconsistent or lacking information on certain disease endpoints.	Requested that several grantees assess the same disease endpoints using different approaches and models to validate data across laboratories and models.
Lack of overlapping endpoints among studies.	Requested that grantees add additional endpoints to studies to provide data on endpoints across laboratories.
Lack of mechanistic studies.	Requested that grantees focus on understanding not only BPA effects but also underlying mechanisms.
Lack of dose response data; lack of understanding of low dose effects and non-monotonic dose responses.	All grantees were asked to assess dose responses, not just single doses of BPA, and to include doses in the μg and ng/kg range.
Lack of understanding of gender differences in BPA effects.	Grantees were asked to assess both sexes.
Lack of understanding of pharmacokinetics across lifespan and species.	Grantees, the pharmacokinetic study, the cashiers study, and the occupational study will assess BPA levels in humans across time and will enable comparisons across species.
Discrepancies between investigator-initiated research results and GLP-compliant studies.	CLARITY study will include five doses of BPA, two doses of ethinyl estradiol, and 12 endpoints assessed by academic researchers.

Supplemental Material, Table S1 (cont.)

Research Challenge	NIEHS Research Investments
Lack of comprehensive assessment of current state of the science.	Consortium working groups are preparing reviews of the literature. Reviews on low dose effects, pharmacokinetics, reproduction, neurobehavior, biomonitoring, and cancer are being developed along with an overall consensus statement.
Questions about the purity and standardization of BPA used in experiments.	NTP provided characterized BPA for use by all grantees and also BPA standard for use in BPA assays.
Lack of coordination of all NIEHS activities.	Trans-NIEHS BPA Working Group established to coordinate activities and share results.

Supplemental Material, Table S2. BPA-focused Grand Opportunity grants supported by funding from the American Recovery and Reinvestment Act (ARRA).

Area of Focus	Principal Investigator	Institution
Mammary cancer	Ana Soto	Tufts University
Prostate cancer	Gail S. Prins, Shuk-Mei Ho, and Kevin P. White	University of Illinois at Chicago; University of Cincinnati; University of Chicago
Prostate cancer	Cheryl L. Walker, Shuk-Mei Ho, and Michael A. Mancini	University of Texas M.D. Anderson Cancer Center; University of Cincinnati; Baylor College of Medicine
Prostate disease	Frederick vom Saal and William Allen Ricke	University of Missouri, Columbia; University of Rochester School of Medicine and Dentistry
Metabolism	Beverly Sharon Rubin and Andrew S. Greenberg	Tufts University
Cardiac function	Scott M. Belcher	University of Cincinnati
Children’s growth and development	Kim Harley and Brenda Eskenazi	University of California, Berkeley
Neurodevelopment and behavior	Shanna H. Swan and Bernard Weiss	University of Rochester
Immune system effects	Robin Marjorie Whyatt	Columbia University Health Sciences
Immune system effects	B. Paige Lawrence	University of Rochester

Supplemental Material, Table S3. BPA research supported by CLARITY study.

**Area of Focus**	**Principal Investigator**	**Institution**
Development of male urogenital system	Frederick vom Saal	University of Missouri
Male reproduction	Kim Boekelheide	Brown University
Male reproduction/sexual function	Nestor Gonzalez-Cadavid	Los Angeles Biomedical Research Institute at Harbor-UCLA Medical Center
Female reproduction	Jodi Flaws	University of Illinois at Urbana-Champaign
Uterine cancer	Shuk-Mei Ho	University of Cincinnati
Prostate cancer	Gail Prins	University of Illinois at Chicago
Mammary cancer	Ana Soto	Tufts University
Metabolism and obesity	Nira Ben-Jonathan	University of Cincinnati
Metabolism and diabetes	Andrew Greenberg	Tufts University
Neurobehavior	Heather Patisaul	North Carolina State University
Brain and thyroid function	Robert Zoeller	University of Massachusetts, Amherst
Immune function	Norbert Kaminski	Michigan State University

Supplemental Material, Table S4. BPA-focused workshops organized by NIEHS.

Description	Date	Location
"Bisphenol A: An Examination of the Relevance of Ecological, In vitro and Laboratory Animal Studies for Assessing Risks to Human Health" (workshop that led to the publication of the "Chapel Hill consensus statement")	November 28-30, 2006	Chapel Hill, NC
Public meetings held by the NTP-CERHR Bisphenol A Expert Panel	March 5-7, 2007<br>August 6-8, 2007	Alexandria, VA
BPA Grantee Consortium workshops	October 6, 2009<br>September 21-22, 2010<br>January 17-19, 2012	Research Triangle Park, NC
CLARITY-BPA workshop	February 29- March 1, 2012	Little Rock, AK
